# A Novel Testing Model for Opportunistic Screening of Pre-Diabetes and Diabetes among U.S. Adults

**DOI:** 10.1371/journal.pone.0120382

**Published:** 2015-03-19

**Authors:** Yurong Zhang, Gang Hu, Lu Zhang, Rachel Mayo, Liwei Chen

**Affiliations:** 1 The First Affiliated Hospital of Medical School, Xi’an Jiaotong University, Xi’an, Shaanxi, China; 2 Pennington Biomedical Research Center, Baton Rouge, LA, United States of America; 3 Epidemiology Program, School of Public Health, Louisiana State University Health Sciences Center, New Orleans, LA, United States of America; 4 Department of Public Health Sciences, Clemson University, Clemson, SC, United States of America; Medical University Innsbruck, AUSTRIA

## Abstract

**Objective:**

The study aim was to evaluate the performance of a novel simultaneous testing model, based on the Finnish Diabetes Risk Score (FINDRISC) and HbA1c, in detecting undiagnosed diabetes and pre-diabetes in Americans.

**Research Design and Methods:**

This cross-sectional analysis included 3,886 men and women (≥ 20 years) without known diabetes from the U.S. National Health and Nutrition Examination Survey (NHANES) 2005-2010. The FINDRISC was developed based on eight variables (age, BMI, waist circumference, use of antihypertensive drug, history of high blood glucose, family history of diabetes, daily physical activity and fruit & vegetable intake). The sensitivity, specificity, and the receiver operating characteristic (ROC) curve of the testing model were calculated for undiagnosed diabetes and pre-diabetes, determined by oral glucose tolerance test (OGTT).

**Results:**

The prevalence of undiagnosed diabetes was 7.0% and 43.1% for pre-diabetes (27.7% for isolated impaired fasting glucose (IFG), 5.1% for impaired glucose tolerance (IGT), and 10.3% for having both IFG and IGT). The sensitivity and specificity of using the HbA1c alone was 24.2% and 99.6% for diabetes (cutoff of ≥6.5%), and 35.2% and 86.4% for pre-diabetes (cutoff of ≥5.7%). The sensitivity and specificity of using the FINDRISC alone (cutoff of ≥9) was 79.1% and 48.6% for diabetes and 60.2% and 61.4% for pre-diabetes. Using the simultaneous testing model with a combination of FINDRISC and HbA1c improved the sensitivity to 84.2% for diabetes and 74.2% for pre-diabetes. The specificity for the simultaneous testing model was 48.4% of diabetes and 53.0% for pre-diabetes.

**Conclusions:**

This simultaneous testing model is a practical and valid tool in diabetes screening in the general U.S. population.

## INTRODUCTION

Diabetes, particularly type 2 diabetes (T2D), is one of the fastest growing public health problems worldwide [1]. In the United States (U.S.), the total number of people with diabetes is projected to increase from 17.7 million in 2000 to 30.3 million in 2030 [1]. Diabetes and its complications remain major causes of morbidity and mortality in the U.S. [2]. The total estimated cost for diabetes in 2012 was $245 billion, 3 a 41% increase from the estimate in 2007 of $174 billion in the U.S. [3]. In addition, approximately 30% of people with diabetes in the U.S. are undiagnosed, and the average lag between onset and diagnosis is 7 years. As many as one quarter of newly diagnosed diabetic patients already have established diabetic retinopathy and/or nephropathy [4,5,6]. Early intervention has been shown to be critical to improve the progression of the complications and reduce the cost of the disease in the long term [7]. Emerging evidence from well-designed randomized controlled trials (RCTs) has demonstrated that the lifestyle intervention with or/without pharmacological treatment can prevent or delay the onset of T2D among people at pre-diabetes stage [8,9]. Therefore, identifying people at pre-diabetes and subclinical stage of T2D is important for public health and clinical perspectives [10].

Historically, routine screening for diabetes in primary practice and the community is challenging. There is no global consensus on the screening strategy and test for detection of diabetes. Fasting plasma glucose (FPG) has been a commonly used tool in screening diabetes, but it has a large random variation, only reflects current glycemic status, and requires people to fast for at least 8 hours before the test. Oral glucose tolerance test (OGTT) has been shown to be the most valid tool for diagnosing diabetes [11]. However, OGTT is more expensive, inconvenient, and has weak reproducibility, making it practically unacceptable for most patients and providers as the first line of screening tool [12]. Glycosylated hemoglobin reflects long-term glycemic control and is a more accurate and stable measure than FPG levels [13,14]. In 2010, the International Expert Committee recommended the use of HbA1C, a non-fasting test, to diagnose diabetes [8]. The recommendations imply that the practical advantages of HbA1c over FPG and OGTT will make diabetes screening more widespread. However, the sensitivity of using HbA1c alone in detecting diabetes is unacceptably low, ranged from 63.2% at a cutoff value of 6.1% to 28.3% at a cutoff value of 7.0% in different populations [15,16].

To our knowledge, 14 diabetes risk score (questionnaires) for screening undiagnosed and/or pre-diabetes have been developed and validated in different populations worldwide [17,18,19,20,21,22,23,24,25]. Some diabetes risk scores (questionnaires) are based on demographic, clinical information and modifiable lifestyle factors such as diet and physical activity, and do not require blood draws and laboratory tests. Therefore, they are cheap and easy to be applied in the primary care setting and in large scope screening programs. However, no studies have been carried out to evaluate whether adding a simple risk score to HbA1C, a clinical routing non-fasting biomarker, would improve the sensitivity in identifying undiagnosed diabetes and pre-diabetes in general U.S. population. Among all the diabetes risk scores (questionnaires) we reviewed, we selected the Finnish Diabetes Risk Score (FINDRISC) because it is: 1) simple (could be administrated by a lay person); 2) non-invasive (no blood draw required); and 3) includes modifiable diabetes risk factor such as diet, physical activity, and body weight. Moreover, FINDRISC is the most commonly evaluated diabetes risk score worldwide [17,18,19,20,21,22,23,24,25] and has been recommended as the best available risk assessment tool for use in the clinical practice by a systematic review [11]. In addition, our group recently evaluated the validity of FINDRISC in the general U.S. population and found it is a valid and reliable tool to identify undiagnosed diabetes and pre-diabetes in both men and women with different races/ethnicities [26]. The objective for the present study was, therefore, to evaluate the performance of a novel simultaneous testing model based on a combination of HbA1c and FINDRISC, which do not require fasting blood samples, in detecting pre-diabetes and undiagnosed diabetes in the general U.S. population.

## RESEARCH DESIGN AND METHODS

### Study Population and Subjects

Subjects for this study were participants of the U.S. National Health and Nutrition Examination Survey (NHANES) 2005–2010. The NHANES 2005–2010 includes three cross-sectional surveys (2005–2006, 2007–2008, and 2009–2010), including national representative samples of non-institutionalized U.S. population. We did not include data before NHANES 2005 because the OGTT tests (with a 75-gram glucose drink) were only added to the NHANES since the 2005–2006 survey. In general, NHANES data were collected through household interviews and health examinations. The household interview included questionnaires on demographic, socioeconomic, dietary, and health-related information. The health examination component consisted of medical, dental, and physiological measurements, as well as laboratory tests administered by trained medical personnel in a fully equipped mobile examination center (MEC). Details of the NHANES laboratory measurement procedures are available at the Center for Disease Control and Prevention (CDC) website. Of particular relevance to the current study, the plasma glucose was measured using a modified hexokinase enzymatic method and HbA1c was measured with the method of high-performance liquid chromatography.

Participants were included in this study if they were aged 20 years or older, attended the morning examination sessions (random selected subsamples were given a fasting blood sample on the morning of their examination), and had fasting for at least 9 hours (but less than 24 hours) (N = 4893). Participants with missing HbA1c (N = 87), FPG (N = 15), OGTT (N = 806), with self-reported diabetes (N = 99), were excluded. The final sample size for the current study was 3886 men and women without diagnosed diabetes. All the data are available at the CDC NHANES website and are open to public access (http://wwwn.cdc.gov/nchs/nhanes/search/nhanes11_12.aspx).

### Finnish Diabetes Risk Score (FINDRISC)

FINDRISC was originally developed in a prospective cohort study in a Finnish population to predict the 10 year diabetes incidence [27]. The FINDRISC is calculated based on eight scored questions about known risk factors for diabetes, including age (years), body mass index (BMI: kg/m^2^), waist circumference (WC: cm), history of antihypertensive drug treatment, history of high blood glucose, family history of diabetes, daily consumption of fruits, berries, or vegetables, and daily physical activity. The final score is the sum of the scores from eight questions, ranging from 0 to 26. In the current study, BMI and WC were identified from the anthropometric data, for which the height, weight and WC were measured by trained personnel in MEC. Daily physical activity time was calculated by adding the minutes spent on physical activity for commute, recreation, and work on average for each day. The consumptions of vegetable, fruit or berries were identified from the dietary dataset and were initially collected through 24-hour food recall method. The answers to all the other questions of the FINDRISC were self-reported, where the age was from the demographic dataset and the others from the questionnaire dataset.

### Diabetes and Pre-diabetes Definition

Diagnosis of diabetes and pre-diabetes were made according to the FPG and 2-h plasma glucose (PG) values of 2013 American Diabetes Association (ADA) criteria [28]. Study participants were categorized into three mutually exclusive groups: normal glucose tolerance (NGT), pre-diabetes, and undiagnosed diabetes. Undiagnosed diabetes group included individuals who actually met the diabetes diagnosis criteria according to the FPG and 2-h plasma glucose (PG) values (FPG ≥126 mg/dl or 2-h PG≥200 mg/dl) but were unknown or never been told they have diabetes [29]. Pre-diabetes group included individuals who had FPG between 100 mg/dl and 125 mg/dl or 2-h PG between 140 mg/dl and 199 mg/dl. Individuals with pre-diabetes were further divided into isolated impaired fasting glucose (IFG), isolated impaired glucose tolerance (IGT), or both IFG and IGT (IFG+IGT). Isolated IFG was defined as having a FPG level ≥100 to <125 mg/dl and a 2-h PG level < 140 mg/dl. Isolated IGT was defined by an FPG level <100 mg/dl and a 2-h PG glucose level ≥140 and <199 mg/dl.

### Statistical Analysis

Descriptive data on study participants’ characteristics were expressed as means (standard deviations (SD)) for continuous variables and percentage (%) for categorical variables. Student’s t-test and one way analysis of variance (ANOVA) were applied to compare continuous variables, and χ^2^ tests were applied to compare categorical variables. The analyses were weighted to account for the complex survey design of NHANES. The receiver operating characteristic (ROC) curves were constructed and the area under the curve (AUC) was used to evaluate the performance of using FINDRISC alone or HbA1c alone in detecting undiagnosed diabetes and pre-diabetes. An AUC = 0.5 indicated the test performed no better than chance and AUC = 1.0 indicated perfect discrimination. Sensitivity (percentage of persons with undiagnosed diabetes or pre-diabetes who had a positive test result), specificity (percentage of persons without undiagnosed diabetes or pre-diabetes who had a negative test result), positive (PV+) and negative (PV-) predictive values were calculated for each unit of FINDRISC score from 5 to 9 and for HbA1C test (considered as positive if ≥6.5% for diabetes and ≥ 5.7% for pre-diabetes), and both methods combined. The optimal cutoff points of FINDRISC in this population were determined by the point with the shortest distance in the ROC curve which was calculated as the square root of [(1-sensitivity)^2^ + (1- specificity)^2^]. When calculating these statistics for pre-diabetes, individuals with diabetes were excluded. Sensitivity analyses were performed by restricting analyses to only individuals aged 45 years or above. Statistical significance was considered at the level of p < 0.05. All the statistical analysis was performed using SAS software, version 9.3 (SAS Institute, Cary, NC).

## RESULTS

**Fig 1:**
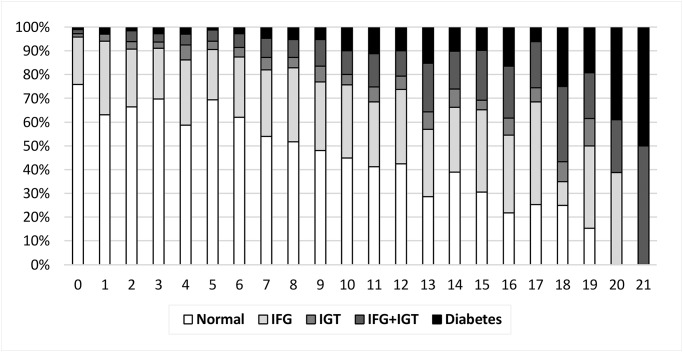
Glucose status at different values of FINDRISC, NHANES 2005–2010.

A total of 3,886 participants aged ≥20 years without self-reported diabetes were included in this analysis. Characteristics of study participants are presented in Table 1. About half of the population were women (47.8%), non-Hispanic white (52.0%), and currently married (55.4%), 24.5% had college degree or above and 13.9% had household income less than $45,000/year. A majority of the population was current alcohol drinkers (76.8%) but were non-smokers (78.9%). Of all participants, 53.8% had normal glucose tolerance (NGT), 41.0% had pre-diabetes (27.1% was isolated IFG, 4.4% was isolated IGT, 9.5% was IFG+IGT), and 5.3% had undiagnosed diabetes. More females were found in the IGT group than in the IFG group (63.2% vs. 33.7%; P < 0.001). The mean HbA1c level was 5.28% in NGT group, 5.47% in isolated IFG group, 5.43% in isolated IGT group, 5.63% in IFG+IGT group, and 6.24% in diabetes group. Mean levels of FINDRISC were increased monotonically, from normal glycemic status to diabetes (7.28 in NGT group, 9.02 in isolated IFG group, 9.79 in isolated IGT group, 11.3 in IFG+IGT group and 11.67 in diabetes group; P < 0.001). The distribution of glycemic status categories across the different FINDRISC values is shown in Fig. 1.

**Table 1:** Characteristics of study participants (≥ 20 years, N = 3,886), NHANES 2005–2010.

	All	Normal	Pre-diabetes			Diabetes	P-value
			IFG	IGT	IFG + IGT		
**N (%)**	3,886 (100.00)	1,938 (53.75)	1,076 (27.08)	199 (4.37)	400 (9.51)	273 (5.29)	
**Age**, year	47.78 (17.00)	41.60 (15.64)	47.87 (15.89)	52.87 (17.72)	57.40 (15.94)	59.23 (15.52)	<0.001
**Female**, %	47.76	56.40	33.74	63.32	41.25	39.93	<0.001
**Race/Ethnicity**, %							
Non-Hispanic white	51.96	51.44	50.19	48.24	59.50	54.21	
Non-Hispanic black	17.19	19.35	17.38	15.58	10.25	12.45	
Hispanics	26.66	25.03	27.97	32.16	25.00	31.50	
Other	4.19	4.18	4.46	4.02	5.25	1.83	0.001
**Annual household income** < $45,000/year, %	13.86	13.23	11.97	21.05	15.64	15.30	0.009
**Marriage status** (married), %	55.40	51.29	59.11	60.30	59.00	61.17	<0.001
**Education** (have college degree or above), %	24.50	27.45	23.70	19.10	21.00	15.75	<0.001
**Current smoking** (yes), %	21.08	22.60	21.47	16.08	17.25	18.01	<0.001
**Current alcohol intake** (yes), %	76.77	77.45	80.63	65.97	69.72	75.00	
**BMI**, kg/m^2^	28.21 (5.96)	26.83 (5.44)	29.11 (5.86)	28.88 (6.05)	30.41 (6.49)	30.69 (6.43)	<0.001
**Waist circumference**, cm	96.16 (14.84)	92.33 (13.67)	100.00 (14.09)	97.57 (14.67)	104.44 (15.30)	105.76 (15.18)	<0.001
**FPG**, mg/dl	100.40 (17.21)	91.43 (5.70)	106.15 (5.04)	93.19 (5.23)	109.26 (6.67)	133.67 (42.64)	<0.001
**2-H PG**, mg/dl	166.87 (48.83)	93.35 (21.80)	104.65 (21.38)	158.35 (14.89)	162.79 (15.74)	234.46 (76.40)	<0.001
**HbA1c**, %	5.44 (0.56)	5.28 (0.35)	5.47 (0.38)	5.43 (0.38)	5.63 (0.38)	6.24 (1.36)	<0.001
**FINDRISC**	8.61 (4.66)	7.28 (4.39)	9.02 (4.56)	9.79 (4.31)	11.32 (4.17)	11.66 (4.16)	<0.001

FINDRISC: Finnish Diabetes Risk Score

FPG: Fasting plasma glucose

PG: Plasma glucose

**Fig 2:**
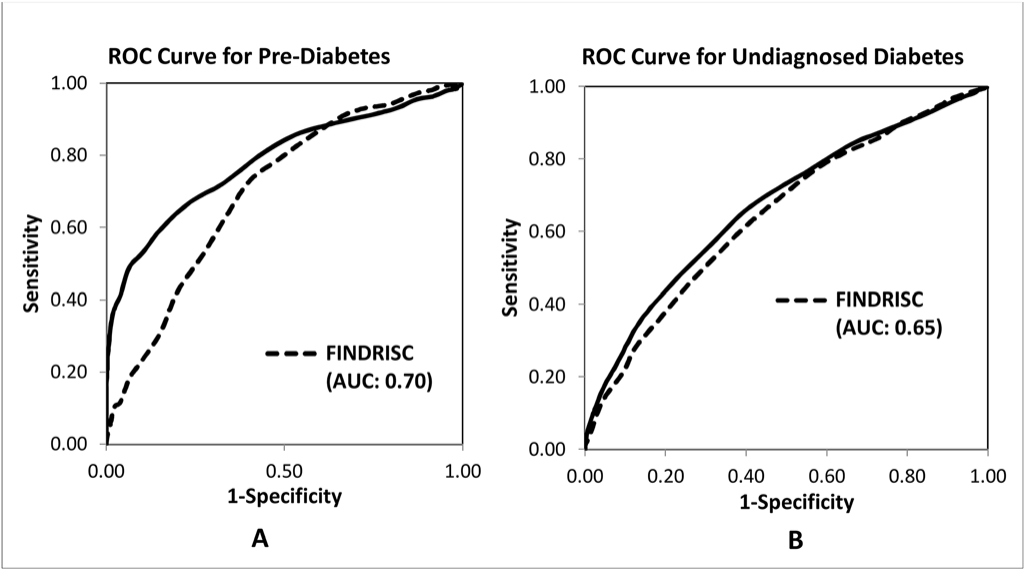
Receiver operating characteristics (ROC) curve for identifying diabetes (A) and pre-diabetes (B) by using FINDRISC and HbA1c in U.S. adults (≥ 20 years), NHANES 2005–2010.

As shown in Table 2, the sensitivity of FINDRISC for identifying undiagnosed diabetes and pre-diabetes decreased as the specificity increased. At the cutoff value of 9, the distance in ROC curve was the shortest for both undiagnosed diabetes (0.53) and pre-diabetes (0.55). The ROC curves for identifying undiagnosed diabetes and pre-diabetes using FINDRISC alone and HbA1C are shown in Fig. 2. The AUC for identifying undiagnosed diabetes was 0.70 for FINDRISC and 0.78 for HbA1C (Fig. 2, A), with the χ^2^ statistics for the difference equal to 14.63 (P < 0.001). For identifying pre-diabetes, the AUC was 0.64 for FINDRISC and 0.67 for HbA1C (Fig. 2, B) with the χ^2^ statistics for the difference equal to 4.53 (P = 0.03).

**Table 2:** Sensitivity and specificity of FINDRISC score in predicting pre-diabetes and diabetes in U.S. adults (≥ 20 years), NHANES 2005–2010.

	Cutoffs	Sensitivity	Specificity	PV+	PV-	Sum	Distance in the ROC	% in the population
**Diabetes**	5	94.14	20.90	8.35	97.72	115.04	0.79	80.16
	6	93.04	27.87	8.88	98.15	120.91	0.72	73.63
	7	90.84	34.87	9.78	98	125.71	0.66	67.83
	8	83.88	44.81	10.3	97.35	128.69	0.57	57.21
	**9**	79.12	51.40	10.95	97.02	130.52	0.53	50.75
**Pre-diabetes**	5	86.03	26.88	50.42	69.1	112.91	0.74	73.55
	6	81.55	35.72	52.42	69.04	117.27	0.67	67.06
	**7**	76.84	43.19	53.85	68.33	120.03	0.61	61.45
	8	66.63	54.70	55.97	65.47	121.33	0.56	51.31
	**9**	60.18	61.40	57.4	64.08	121.58	0.55	45.19

PV+: Positive predict value

PV-: Negative predict value

Sum: Sensitivity + Specificity

**Fig 3:**
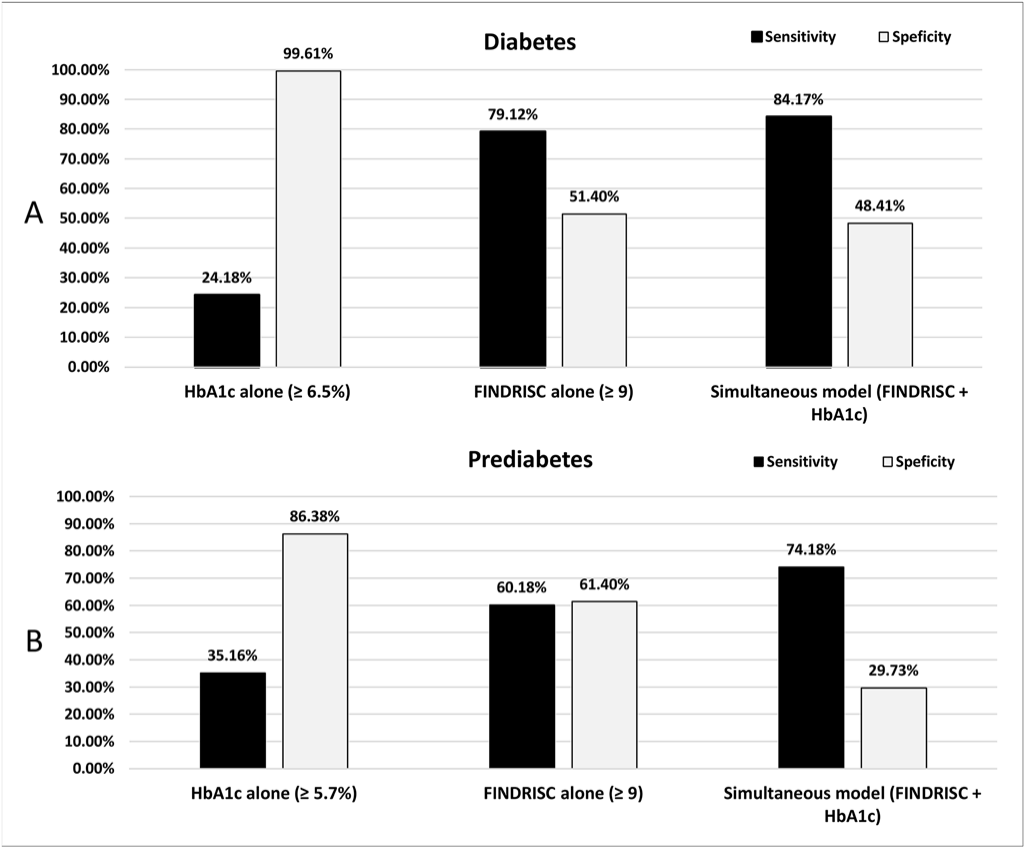
Sensitivity and specificity for detecting undiagnosed diabetes (A) and pre-diabetes (B) in U.S. adults (≥ 20 years) by HbA1c alone, FINDRISC alone, and the new simultaneous model.

For identifying undiagnosed diabetes, using HbA1c ≥6.5% alone had an excellent specificity (99.61%), but very poor sensitivity (24.2%). The sensitivity and specificity for using HbA1c ≥5.7% alone to identify pre-diabetes (isolated IFG, isolated IGT, or IFG+IGT) was also poor: 35.2% and 86.4% (with 31.5% and 77.9% for isolated IFG, 31.2% and 76.3% for isolated IGT), respectively. Using the FINDRISC alone, the sensitivity and specificity was 60.2% and 61.4% for pre-diabetes (with 54.4% and 52.2% for isolated IFG, 61.8% and 51.8% for isolated IGT) and was 79.1% and 51.4% for undiagnosed diabetes, respectively. When HbA1c and FINDRISC were combined together to create a simultaneous test model (a person was considered as positive, if he/she tested positive on either test), the sensitivity was substantially improved to 84.2% for diabetes and 74.2% for pre-diabetes (68.3% for isolated IFG and 73.7% for isolated IGT) (Fig. 3); the specificity was 48.4% of diabetes and 53.0% for pre-diabetes; and the AUC for diabetes was 0.80 for diabetes (χ^2^ = 33.0; P < 0.001) and 0.70 for pre-diabetes (χ^2^ = 56.9; P < 0.001).

We performed the sensitivity analysis by conducting the above analyses only among participants aged 45 years or above (N = 2,026) and the results did not change (data not shown). For example, among individuals ≥ 45 years: the sensitivity and specificity were 43.6% and 76.4% for using HbA1c ≥5.7% alone to identify pre-diabetes; the sensitivity and specificity were 24.2% and 99.6% for using HbA1c ≥6.5% alone to identify diabetes. Using FINDRISC alone among individuals ≥ 45 years: the sensitivity and specificity were 73.2% and 42.5% to identify pre-diabetes; the sensitivity and specificity were 87.0% and 33.4% to identify diabetes. When HbA1c and FINDRISC were combined together to create a simultaneous test model, the sensitivity was substantially improved to 90.1% for diabetes and 84.9% for pre-diabetes.

## DISCUSSION

Although diabetes scores and HbA1c have both been recommended for screening for diabetes, the present study shows that using each method alone does not have sufficient sensitivity to screen for both pre-diabetes and undiagnosed diabetes in a general adult population in the U.S. The sensitivity for HbA1c alone was only 24.18% for diabetes (HbA1c ≥6.5%) and 35.16% for pre-diabetes (HbA1c ≥5.7%) which were even lower than 50%, indicating the test actually performed worse than just flipping a coin in identifying individuals with diabetes and pre-diabetes. However, using the simultaneous testing model with combination of FINDRISC and HbA1c improved the sensitivity to 74.18% to identify pre-diabetes and to 84.17% to identify undiagnosed diabetes, suggesting it is a practical and sufficient tool in diabetes screening in the U.S. population.

Diabetes is a huge global health problem and undiagnosed diabetes exacerbates the situation. The true challenge is to identify asymptomatic individuals in typical opportunistic settings (i.e. screening patients who visit a health care provider for health problems other than diabetes) and community health programs. A number of organizations have advocated for opportunistic diabetes screening of people presenting to primary care as a critical strategy to reduce the disease burden (Table 3) [30,31,32,33,34,35]. However, questions remain about the optimal screening methods and cutoff points for a positive test. FPG and OGTT both require people to be fasting, which is difficult to perform in large populations for screening purposes and is inconvenient in routine clinical 4practice. HbA1c, a non-fasting test, has the practical advantages over FPG and OGTT and is not affected by short-term lifestyle changes (day-to-day variation less than 2%). HbA1c is familiar to clinicians and is widely available in the U.S. Currently, over 99% of laboratories in the U.S. measuring the HbA1c use the National Glycohemoglobin Standardization Program (NGSP) certified-methods. A survey conducted among U.S. physicians in 2005 showed that 93.4% of them routinely screen for diabetes, 49% using the HbA1c for screening and 58% for diagnosis [36]. However, the problem is the sensitivity of using HbA1c alone in detecting diabetes is unacceptably low. As shown in Table 4, the sensitivity of using HbA1c alone in detecting diabetes ranged from 78.7% at a cutoff value of 5.3% to 16.7% at a cutoff value of 6.1% in different populations. Two previous studies also examined the performance of using HbA1c in screening for undiagnosed diabetes in the U.S. population, using the NHANES data [15,16]. In the study conducted by Rohlfing et al. (NHANES III), the sensitivity was 42.8% using HbA1c at a cutoff value of 6.5% [16]. Similar results were reported by Buell et al. by using the NHANES 1999–2004 [15]. In the present study, we found that the sensitivity of using the HbA1c alone was just 24.0% to identify undiagnosed diabetes (cutoff of ≥ 6.5%) and 35.0% to identify pre-diabetes (cutoff of ≥ 5.7%). Different from the previous two studies which only used FPG as a reference standard to diagnose diabetes; we used both FPG and 2-h PG as the reference standard. This approach allowed us to identify people with abnormal postprandial glucose level and with IGT. In addition, our study reported new information on the performance of using HbA1c in identifying pre-diabetes, including isolated IFG, isolated IGT, and IFG+IGT. Despite these differences, our study is in agreement with the other two studies showing that the HbA1C test alone is not a sufficient method for screening diabetes and pre-diabetes in primary and community settings.

**Table 3:** Current Guidelines for Diabetes Screening.

Organization [reference]	Recommendations
AACE, 2011 [30]	• All individuals aged ≥30 years who meet any of the clinical risk criteria noted below should be screened for pre-diabetes or Type 2 diabetes. Testing should be considered in all adults who are overweight (BMI ≥25 kg/m2) and who have ≥1 of the additional risk factors [i.e. Physical inactivity; First-degree relative with diabetes; High-risk race/ethnicity (eg, African American, Latino, Native American, Asian American, Pacific Islander); Hypertension (blood pressure>140/90 mmHg or on therapy for hypertension); HDL cholesterol level 250 mg/dL (2.82 mmol/L); A1C ≥5.5%, IGT, IFG, or metabolic syndrome on previous testing; Delivered a baby weighing>9 pounds or patient was previously diagnosed with gestational diabetes mellitus (women); Polycystic ovary syndrome (PCOS) (women); Other clinical conditions associated with insulin resistance (eg, severe obesity, acanthosis nigricans); History of CVD; Smoking).
	• If a patient does not meet any of the above criteria, testing for T2DM should begin at age 45 years. In the event of normal results, repeat testing at least every 3 years.
ADA, 2014 [31]	• Testing to detect type 2 diabetes and pre-diabetes in asymptomatic people should be considered in adults of any age who are overweight or obese (BMI ≥25 kg/m2) and who have one or more additional risk factors for diabetes.
	• In those without these risk factors, testing should begin at age 45 years.
	• If tests are normal, repeat testing at least at 3-year intervals is reasonable.
	• To test for diabetes or pre-diabetes, the A1C, FPG, or 2-h 75-g OGTT are appropriate (A1C≥6.5%; FPG ≥7.0 mmol/L; 2-h PG in 75-g OGTT ≥11.1 mmol/L).
	• In those identified with pre-diabetes, identify and, if appropriate, treat other CVD risk factors.
ANHMRC, 2009 [32]	• A three-step case detection and diagnosis procedure is recommended for detecting people with undiagnosed type 2 diabetes:
	1. Initial risk assessment determined using a risk assessment tool or risk factors commonly associated with undiagnosed type 2 diabetes
	2. Measurement of fasting plasma glucose
	3. An oral glucose tolerance test performed in all people with an equivocal result: FPG of 5.5–6.9 mmol/L, or random plasma glucose of 5.5–11.0 mmol/L.
	• Periodic re-testing for undiagnosed type 2 diabetes is recommended according to the following schedule: each year for people with impaired glucose tolerance or impaired fasting glucose; every 3 years for all other people.
	Screening for undiagnosed type 2 diabetes in high risk individuals should be an integral component of a diabetes prevention program.
CTFPHC, 2012 [33]	• To assess risk level among the general population, use a validated risk calculator rather than a routine blood test for the first stage of screening. The preferred screening tool is the Finnish Diabetes Risk Score (FINDRISC), although the Canadian Diabetes Risk Assessment Questionnaire (CANRISK) is an acceptable alternative. These validated risk calculators may also educate patients about their risk factors.
	• The preferred blood test for screening is A1C, although fasting glucose measurement and the oral glucose tolerance test are acceptable alternatives. The recommended threshold for diagnosing diabetes is an A1C level of at least 6.5%, but lower values do not exclude diabetes diagnosed with glucose tests. A1C should be measured with a standardized, validated assay.
	• Perform A1C blood test screening every 3 to 5 years among adults who are at least 40 years of age and at low to moderate risk and among those at high risk at any age. Perform A1C blood test screening annually among adults of any age who are at very high risk for diabetes.
USPSTF, 2008 [34]	• Screening for type 2 diabetes in asymptomatic adults with sustained blood pressure (either treated or untreated) greater than 135/80 mm Hg
	• The current evidence is insufficient to assess the balance of benefits and harms of routine screening for type 2 diabetes in asymptomatic adults with blood pressure of 135/80 mm Hg or lower
IDF, 2006 [35]	• Universal screening for undiagnosed diabetes is not recommended. Detection programs should target high-risk peopleidentified by assessment of risk factors.
	• Detection programs should use measurement of plasma glucose, preferably fasting.
	• Where a random plasma glucose level ≥ 5.6 mmol/ l (≥100 mg/dl) and < 11.1 mmol/ l (< 200 mg/dl) is detected on opportunistic screening, it should be repeated fasting, or an OGTT performed.
	• People with screen-detected diabetes should be offered treatment and care.

AACE = American Association of Clinical Endocrinologists

ACOG = American College of Obstetricians and Gynecologists

ADA = American Diabetes Association

ANHMRC = Australia National Health and Medical Research Council

CTFPHC = Canadian Task Force on Preventive Health Care

IDF = International Diabetes Federation

USPSTF = U.S. Preventive Services Task Force

IFG = impaired fasting glucose; IGT = impaired glucose tolerance; OGTT = oral glucose tolerance test;

**Table 4:** Literature on the sensitivity and specificity of screening test using HbA1c in general populations.

Study (First author, year, reference)	Country	Sample Size	Age Range	Screening Test	Sensitivity	Specificity	Gold Standard Test
**Pre-diabetes**							
Lee, 2013 [45]	Korea	4,616	>18 y	HbA1c (≥5.7%)	48.6%	65.7%	OGTT
Colagiuri, 2004 [38]	Australia	10,477	>25 y	HbA1c (≥5.3%)	42.0%	88.2%	OGTT
Mannucci, 2003 [43]	Italy	1,215	30–70 y	HbA1c (>5.5%) or FPG (>6.1 mmol/l)	59.0% (M) 54.8% (F)	19.3% (M) 9.3% (F)	OGTT
Saydan, 2002 [44]	US	2,844	40–74y	FPG (≥6.1 mmol/l) or HbA1c (≥5.5%)	45.5%	81.3%	OGTT
Saydan, 2002 [44]	US	2,844	40–74y	HbA1c (≥6.0%)	16.7%	92.9%	OGTT
**Diabetes**							
Buell, 2007 [15]	US	4,935	≥20 y	HbA1c (≥5.8%)	86.0%	92.0%	FPG
Nakagami, 2007 [41]	Japan	1,904	35–89 y	HbA1c (≥5.6%)	56.5%	96.1%	FPG
Droumaguet, 2006 [42]	France	2,820	30–65 y	HbA1c (≥6.3%)	77.0%	86.0%	FPG
Colagiuri, 2004 [38]	Australia	10,447	>25 y	HbA1c (≥5.3%) and RF	78.7%	82.8%	OGTT

RF: risk factor

“And”: two tests were conducted sequentially

“Or”: two tests were conducted simultaneously

There are two common ways to improve the sensitivity for a given test method. The first way is to use a lower cutoff value. For HbA1c, studies have shown that lowering the cutoff value from 6.5% to 5.6% substantially increased the sensitivity from 42.3% to 83.4% in U.S. population [16]. However, such an approach will result in a larger number of false positives (people who are considered as having positive rests but do not actually have the disease) which would be costly but inefficient, and put a huge burden on the health care system. In addition, it is well known that the HbA1c test is not effective in detecting IGT [37]. Even lowering the cutoff value to 5.3%, resulted in a sensitivity of only 78% among individuals with at least one risk factor in a study conducted in Australia national population [38]. From the perspective of natural history of T2D, postprandial hyperglycemia is one of the earliest biomarkers for disease progression. Therefore, IGT indicates a critical window of early effective intervention and this has been shown by several RCTs across different populations and racial groups. However, lowering the cutoff value of the HbA1c test will not be able to overcome this drawback.

The second way to increase sensitivity of a given test method is to combine it with another test. Screening tests may be combined simultaneously (i.e. a person is considered to be positive if he/she tests positive to *either* test) or sequentially (i.e. a person is considered to be positive he/she tests positive in *both* tests). In the case of simultaneous testing, the sensitivity and the negative predictive value are generally increased and the specificity and positive predictive values decreased. On the other hand, if sequential testing is applied, the specificity and positive predictive value are generally increased and the sensitivity and negative predicted value decreased. Tests in series have been advocated in T2D by a few of professional organizations such as ADA and American Association of Clinical Endocrinologists (AACE) (Table 3). Most of them recommended a two-stage strategy, starting with the first-stage of selection of people at higher risk by a questionnaire or risk score and followed by the second-stage of testing for conventional biomarkers such as FPG, HbA1c, or OGTT [12,39]. The rationale for such a recommendation is that there is no evidence from RCT to show that universal screening of asymptomatic individuals will be effective or cost-effective in improving diabetic complications or mortality. However, a recent large cluster RCT from the UK reported that screening for diabetes among individuals at increased risk (based on a diabetes risk score) was not associated with a lower rate of all cause or CVD mortality within 10 years [40]. In this study, a sequential testing was applied and only those who had risk scores greater than a certain value (considered as high-risk individuals) were invited for additional laboratory tests. Moreover, using two tests sequentially will generally increase the specificity but reduce the sensitivity, which will result in a substantial number of individuals with diabetes unidentified. In other words, theoretically such an approach will not help to solve the current high rate of undiagnosed diabetes.

In the present study, the simultaneous testing model, based on the combination of a simple diabetes risk score and HbA1c test, is a practical and reliable tool in diabetes screening in the U.S. population. There are several notable advantages of this study. First, the present study was conducted in a large sample of U.S. representative adults with unknown diabetes. Second, the 75-g glucose OGTT test was used as the reference standard for diagnosing and the test model was evaluated for diabetes and pre-diabetes, including isolated IFG, isolated IGT, and IFG+IGT. Third, the selected FINDRISC score was developed based on individual’s information on eight questions that do not require any laboratory tests. In addition, our previous study has indicated that FINDRISC score is a valid tool in detecting both undiagnosed diabetes and pre-diabetes in U.S. men and women with different race/ethnicities [26]. Moreover, the HbA1C test does not require fasting and can be conducted at any time of the day. Therefore, the proposed simultaneous model can be used in an opportunistic screening in outpatient visits or community screening programs. Our study is limited in that each laboratory test (i.e. HbA1c, FPG, and OGTT) was only tested for once in NHANES 2005–2010. Although previous studies have reported that both FPG and OGTT have a day-to-day variation (within person variability) of 12–17% [4], OGTT is still the best reliable test for diabetes and has been considered as “gold standard” test in evaluating other tests. HbA1c, on the other hand, has a very small day-to-day variation (<2%). Therefore, the possibility of misclassification, if it exists, would be small in the present study. We applied the current ADA-recommended HbA1c cutoff points for diabetes and pre-diabetes. The optimal cutoff values for HbA1c has been intensively reported in previous studies and discussed in several review papers [4,14,15,38,41,42,43,44,45]. Thus, the current study didn’t aim to investigate on using different HbA1c cutoff values. It’s well known that lower the cutoff values of a given test will result in an increase in sensitivity. Therefore, there is no doubt that adding FINDRISC will improve the sensitivity at any given HbA1c value.

## CONCLUSIONS

In summary, our study demonstrates the simultaneous testing model, combining a simple diabetes risk score with the HbA1c test to significantly improve the sensitivity in detecting undiagnosed diabetes and pre-diabetes, including isolated IGT. This model is a simple, practical and reliable tool in opportunistic diabetes screening in the U.S. population. Further study is warranted to evaluate the cost effectiveness of this screening model.
